# Can Real-time Computer-Aided Detection Systems Diminish the Risk of Postcolonoscopy Colorectal Cancer?

**DOI:** 10.2196/25328

**Published:** 2021-12-24

**Authors:** Mariusz Madalinski, Roger Prudham

**Affiliations:** 1 Northern Care Alliance Royal Oldham Hospital Oldham United Kingdom; 2 Northern Care Alliance Bury United Kingdom

**Keywords:** artificial intelligence, colonoscopy, adenoma, real-time computer-aided detection, colonic polyp

## Abstract

The adenoma detection rate is the constant subject of research and the main marker of quality in bowel cancer screening. However, by improving the quality of endoscopy via artificial intelligence methods, all polyps, including those with the potential for malignancy, can be removed, thereby reducing interval colorectal cancer rates. As such, the removal of all polyps may become the best marker of endoscopy quality. Thus, we present a viewpoint on integrating the computer-aided detection (CADe) of polyps with high-accuracy, real-time colonoscopy to challenge quality improvements in the performance of colonoscopy. Colonoscopy for bowel cancer screening involving the integration of a deep learning methodology (ie, integrating artificial intelligence with CADe systems) has been assessed in an effort to increase the adenoma detection rate. In this viewpoint, a few studies are described, and their results show that CADe systems are able to increase screening sensitivity. The detection of adenomatous polyps, which are associated with a potential risk of progression to colorectal cancer, and their removal are expected to reduce cancer incidence and mortality rates. However, so far, artificial intelligence methods do not increase the detection of cancer or large adenomatous polyps but contribute to the detection of small precancerous polyps.

## Introduction

Adenomatous polyps are associated with a potential risk of progression to colorectal cancer (CRC). The adenoma detection rate (ADR) is regarded as an important marker of the quality of inspection in colonoscopy. The identification and removal of adenomatous polyps are considered to be important in CRC prevention [1,2]. More recently, computer-aided detection (CADe) tools that incorporate a 3D fully convolutional network have been developed to aid with colonoscopy screening for CRC. Deep learning methodologies, whereby a programmer teaches a computer which features to focus on, have been developed, thus allowing artificial intelligence (AI) to be integrated during colonoscopy [3,4].

## CADe Tools for Colonic Cancer: The Studies

Repici et al [3] have presented results on their evaluation of the efficacy of integrating the CADe of colonic polyps with high-accuracy, real-time colonoscopy. This provides a unique opportunity to obtain real-time feedback for informing an endoscopist about the quality of a live endoscopy.

In Repici et al’s [3] study, 685 individuals were randomized, and the authors reported a significantly higher ADR in the CADe group. This appears to confirm the findings of Wang et al [4], who enrolled 1058 patients into their first prospective randomized controlled trial. Both studies reported a significantly higher mean number of adenomas and nonpolypoid lesion detection rate in the CADe group than those in the control group [3,4].

A real-time automatic detection system that uses deep neural networks was trialed in Italy, and it achieved a high ADR (CADe group: 54.8%; control group: 40.4%) [3]. However, a much lower ADR was reported for both groups (CADe group: 29.1%; control group: 20.3%) in Wang et al’s [4] study, but the mean age of the participants in this Chinese study was 49.94 years (SD 13.79 years) in the control group and 51.07 years (SD 13.15 years) in the CADe group [4]. This may also be explained by the observation that the overall prevalence of adenomas and CRC is lower in mainland China than in Europe and the United States [5]. Comparing these studies is difficult however, as in the Repici et al [3] study, the patients’ mean age was considerably higher (mean 61.32 years, SD 10.2 years). In their study, a significantly higher number of diminutive adenomas and adenomas that were 6 to 9 mm in diameter were detected in the CADe group, regardless of the adenomas’ location or morphology [3]. In the Wang et al [4] study, CADe helped to significantly increase the detection of adenomas in colonic segments (ie, from the hepatic flexure to the rectosigmoid junction), but the CADe technology appeared to be the most effective at detecting adenomas in the transverse colon. A further analysis revealed that the higher ADR in the CADe group was mainly due to an increase in the detection of diminutive adenomas; there were no significant differences among large ADRs [4].

Recently, a Chinese cross-sectional study [5] reported a higher ADR for the proximal colon compared to that for the distal colon, but this difference was not observed in the Wang et al study [4]. However, this difference was observed by Repici et al’s [3] team. The ratio of precancerous polyps located in the proximal colon to precancerous polyps in the distal colon is another suggested measure of performance that may be used to confirm the high quality of a clearing colonoscopy [6].

## Repici et al’s [3] Study Limitations

The six experienced endoscopists in the Repici et al [3] study had over 2000 screening colonoscopies under their belts. We do not know if more experienced endoscopists—those who have performed more than 10,000 colonoscopies—would confirm Repici et al’s [3] results. Moreover, the endoscopists were required to adhere to a minimum of 6 minutes for inspection; their mean withdrawal time was around 7 minutes [3] (the withdrawal time was a little shorter in Wang et al’s [4] study).

The endoscopists’ withdrawal techniques did not meet the criteria for aspirational withdrawal time (≥10 minutes) that are present in the European Society for Gastroenterology guidelines [1] and the British Society of Gastroenterology guidelines [2]. There is evidence that a shorter withdrawal time is associated with a lower ADR and a higher incidence of postcolonoscopy CRC and that a longer withdrawal time increases the ADR [1,2]. The exact mechanism by which withdrawal time impacts the risk of postcolonoscopy CRC and its impacts on the ADR are not well known, but we can hypothesize that withdrawal time affects careful colonic mucosal inspection.

## The Future of Bowel Cancer Screening

Endoscopists’ withdrawal techniques and specified right colon withdrawal times correlate with higher levels of polyp detection [7]. Therefore, a considerable challenge lies ahead of those who wish to use the detection all polyps (via AI methods) as a new independent marker. Further research is needed to determine whether this marker is more optimal than the advised aspirational withdrawal time (≥10 minutes) in current colonoscopy guidelines or the ADR. Additionally, other interesting questions that have arisen are whether the withdrawal time is a better marker than the ADR and whether these markers are surrogate markers for the detection of all polyps that are monitored via AI. Originally, the ADR was defined as the percentage of patients aged ≥50 years who underwent primary screening colonoscopy for the first time and had 1 or more conventional adenomas [1,2].

The adenoma miss rate varies among endoscopists who achieve the same ADRs, and a significant difference in adenoma miss rates has been reported even among endoscopists who achieve high ADRs [8]. A reduction in the number of all colonic adenomas may be recognized as a complementary benchmark of cancer protection after clearing colonoscopies. Therefore, we assume that the removal of all polyps with the potential for carcinogenesis comprises an independent marker of quality that is relevant to clearing colonoscopies, and AI may be helpful for assessing this goal. Thus, as a support for endoscopists who have not developed the highest quality skills, AI creates a new opportunity, especially after the end of colonoscopy training.

Further studies are required to determine whether AI is of benefit to endoscopists who are more experienced than those in Repici et al’s [3] study. Our personal experience reveals that using AI results in the increased incidence of the overdiagnosis of polyps with little or no malignant potential. It is important to not accept as a given that the utility offered by AI-assisted colonoscopy in detecting diminutive polyps is of definite value overall. It is possible that as AI-assisted colonoscopy increases the number of diminutive polyps that are detected, the time taken to complete a colonoscopy also increases, as these polyps must be inspected and removed. This may in turn increase the costs associated with colonoscopy. Within health economies that are constrained by limited resources, AI-assisted colonoscopy may have the unintended consequence of reducing the amount of benefits that are provided to the population as a whole by reducing access to colonoscopy. Long-term outcome studies must be conducted to determine how beneficial this new technology may be, regardless of how exciting it appears to be at first glance.

## Conclusion

So far, we know that AI methods do not increase the detection of large adenomas or cancer. The contribution of small adenomas, which have been increasingly detected via AI-assisted colonoscopy, to future CRC risk is debatable.

## Abbreviations

ADR: adenoma detection rate
AI: artificial intelligence
CADe: computer-aided detection
CRC: colorectal cancer
